# Adenoid Cystic Carcinoma of the Minor Salivary Glands: A Systematic Review and Meta-Analysis of Clinical Characteristics and Management Strategies

**DOI:** 10.3390/jcm13010267

**Published:** 2024-01-03

**Authors:** Mohamed A. Jaber, Mawada Hassan, Mohamed Ingafou, Alaa Mohamed Elameen

**Affiliations:** 1 Department of Clinical Sciences, College of Dentistry, Ajman University, Ajman P.O. Box 346, United Arab Emirates; m.abdelmagied@ajman.ac.ae (M.H.); m.ingagou@ajman.ac.ae (M.I.); 2 Center of Medical and Bio Allied Health Sciences Research, Ajman University, Ajman P.O. Box 346, United Arab Emirates; 3 College of Science, UAE University, Alain P.O. Box 15551, United Arab Emirates; alaajabberr@gmail.com

**Keywords:** adenoid cystic carcinoma, minor salivary glands, head and neck tumor, chemotherapy and radiotherapy of the head and neck

## Abstract

Background: Intraoral adenoid cystic carcinoma (ACC) arising from minor salivary glands (MSG) is a rare malignancy associated with delayed diagnosis and unfavorable outcomes. This study aimed to comprehensively review ACC of MSGs, focusing on clinical characteristics, imaging modalities, treatment approaches, and long-term outcomes. Methods: A systematic search was conducted in PubMed, Web of Science, and MEDLINE databases to identify relevant articles reporting cases of ACC of MSGs between January 1997 and March 2023. The study was registered in PROSPERO (ID: CRD42023449478). A total of 10 studies that met the inclusion criteria were selected for critical review. In total, 902 patients were diagnosed with ACC of MSGs with an age range of 44.3 to 63 years, and an average age of 56.6 years. The female to male ratio ranges from 1:1 to 2.4:1. Regarding the primary site of ACC, the palate was the most common location, accounting for 30.5% to 83.3%, followed by the buccal mucosa, floor of the mouth, and lip and the retromolar area. For histology, the solid mass pattern was the most prevalent, seen in 95.2% of patients, followed by the cribriform pattern. Regarding treatment modalities, surgery was the most common approach, applied in 76.3% of cases, with a combination of surgery and radiotherapy used in 29.0% of cases. A smaller fraction, 3.2%, received a combination of surgery, chemotherapy, and radiotherapy, and 8.3% underwent radiotherapy alone. Local recurrence rates varied between 1% and 28.5%, and distant metastasis occurred in 18.2% to 33.3% of cases, predominantly to lymph nodes (14.5%). An analysis of overall survival across various stages and patient numbers indicated a 5-year survival rate of 68.0%. The findings of this study provide valuable insights for physicians in making treatment decisions and emphasize the need for ongoing research and collaborative clinical efforts to improve the management and outcomes of this challenging disease. Conclusion: ACC of MSGs is a multifaceted condition typically manifesting as asymptomatic enlargement and ulceration. This disease is marked by distinct histopathological patterns and perineural invasion (PNI). Recognizing these distinctive aspects is key in shaping the treatment plan, which can range from surgical procedures to radiation therapy, chemotherapy, and evolving targeted treatments. Continuous research and collaborative clinical efforts remain critical for ongoing progress in the treatment and management of this challenging condition.

## 1. Introduction

Adenoid cystic carcinoma was first described by Billroth in 1859 and characterized by its unique combination of connective and epithelial tissue. ACC is known for its slow progression, local recurrence, and regional and systemic metastasis [1]. ACC represents approximately 1% of all head and neck cancers and 10% of salivary gland tumors. ACC arising from minor salivary glands is rare, comprising only 10–20% of all ACC cases, and it exhibits a high propensity for PNI, contributing to its spread [2].

ACC primarily affects individuals aged 40 to 60 years, with a slight female predominance [3,4]. The most common sites within the oral cavity include the palate, tongue, and floor of the mouth, with additional occurrences in the lips, buccal mucosa, retromolar trigone, and tonsillar area [4,5,6].

The exact etiology of ACC remains unknown. However, recent studies have shed light on molecular abnormalities associated with ACC development and progression. The most common genetic abnormalities involve translocations of the myeloblastosis (MYB) gene on chromosome 6q and the Nuclear factor 1 B (NFIB) gene on chromosome 9p. These translocations result in the overexpression of MYB and NFIB, which play crucial roles in cell growth, differentiation, and survival regulation [7,8].

ACC arising from minor salivary glands typically presents as a painless, slow-growing mass. Patients may also experience numbness or tingling in the affected area due to perineural invasion, which is a characteristic feature of ACC. Clinical presentation may resemble other salivary gland tumors, such as pleomorphic adenoma and mucoepidermoid carcinoma, making biopsy essential for accurate diagnosis [9,10].

Histological examination of a biopsy specimen is necessary for the definitive diagnosis of ACC. The typical histological features of ACC include a cribriform or tubular pattern, with hyaline or basophilic material within the cribriform spaces. ACC comprises two distinct cell types: ductal and myoepithelial cells. Immunohistochemical stains, such as S100 and smooth muscle actin, aid in identifying these cell types and differentiating ACC from other salivary gland tumors [11,12,13]. Differential diagnosis includes benign and malignant tumors, such as pleomorphic adenoma, polymorphic adenocarcinoma, and mucoepidermoid carcinoma.

There is no universally recommended treatment for ACC, highlighting the need for personalized treatment plans based on clinical and histological factors to achieve optimal outcomes. Treatment approaches are typically multidisciplinary and may involve surgery, radiotherapy, chemotherapy, or various combinations thereof. Combined therapies, such as surgery with radiotherapy or radiotherapy with chemotherapy, are often favored [14,15,16]. The prognosis for ACC arising from minor salivary glands is generally poor, with a 5-year survival rate of 50–60%. Prognosis is influenced by factors such as tumor location, size, PNI, and histological grade. Previous reports indicated that ACC with a solid pattern carries a worse prognosis compared to the cribriform pattern, and high-grade transformation is associated with poorer outcomes [17,18].

Due to the rarity of ACC, single studies often lack the comprehensive data needed for definitive findings. The infrequency of ACC cases contributes to the limitations of individual studies, emphasizing the importance of an exhaustive review that amalgamates data from various studies. Additionally, ACC is characterized by a diverse range of clinical symptoms and treatment strategies, coupled with an absence of consensus on certain aspects of its management and clinical presentation. Consequently, a systematic review (SR) and meta-analysis (MA) can methodically compile and scrutinize these diverse elements, enhancing our understanding of ACC’s clinical manifestations. Such reviews not only provide a deeper insight into the disease but also establish a consolidated resource for future research, and identify areas that warrant further exploration; thus, the objectives of this study were to evaluate the epidemiological aspects of ACC arising from minor salivary glands, explore different therapeutic options, and assess survival rates among patients with this tumor.

## 2. Materials and Methods

### 2.1. Protocol and Registration

The protocol was developed in accordance with the Preferred Reporting Items for Systematic Reviews and Meta-Analysis Protocols (PRISMA-P) [19] and registered in PROSPERO (ID: CRD42023449478).

### 2.2. Information Sources and Search Strategy

A comprehensive literature search was conducted to identify relevant studies using various databases, including PubMed/MEDLINE, EMBASE, Cochrane databases, Scopus, Web of Science, and Google Scholar. These were used to identify studies published in the English language between January 1997 and March 2023. Additionally, a manual search of reference lists from retrieved articles and relevant journals was performed. Medical Subject Headings (MeSH) were employed, including “Adenoid cystic carcinoma” OR “Minor salivary gland tumor” and “Clinical manifestation” OR “Characteristics” OR “Symptoms” OR “Head and neck radiotherapy” OR “Chemotherapy” OR “Surgery.” In this systematic review, the PICO encompassed Population (male/female patients), Interventions (surgery, radiotherapy, chemotherapy), Comparisons between different treatment modalities, and Outcomes of interest (prevalence of ACC of MSGs, recurrence, metastasis).

Based on the detailed search strategy and PICO framework outlined, the specific research question for this systematic review was: “How do clinical characteristics and different treatment modalities (surgery, radiotherapy, chemotherapy) compare in terms of effectiveness and outcomes (prevalence of ACC, recurrence, metastasis) in both male and female patients diagnosed with ACC of MSGs?”

### 2.3. Eligibility Criteria

#### 2.3.1. Inclusion Criteria

Studies needed to be available as full-text articles and only in the English language. Moreover, selection was categorized on original studies including retrospective studies, prospective studies, studies containing at least one clinically diagnosed case of ACC of minor salivary glands, studies with a follow-up period of more than 12 months, and studies evaluating the treatment and patient survival.

#### 2.3.2. Exclusion Criteria

Exclusion criteria included non-English articles, non-original articles, duplicate publications (risk of bias), articles without complete demographic information of each patient, editorial letters, case reports, review articles, and studies with a follow-up lower than 12 months.

### 2.4. Data Extraction

Studies were initially selected based on the title and abstract information. Next, the selected articles were read in full text to check for their eligibility. A list of excluded studies was maintained to avoid selection bias between two of the authors (M.H. and E.A.) who reviewed independently. In the case of disagreement between the authors, a consensus was attained. Data extracted included author(s) and year of publication, studied population, sample size, age range of the sample, and study design; gender: the patient’s gender was determined in terms of percentage and ratio, intraoral site of the tumor; histological features: dividing it into cribriform, tubular, and solid type, and linked to the outcome; therapy: the therapeutic approaches used on different patients were analyzed, classified into either surgery or surgery combined with radiation therapy, surgery combined with radiotherapy and chemotherapy, radiation therapy, or chemotherapy; survival analysis: perineural; metastasis: local and/or distant metastasis.

### 2.5. Methodological Assessment of Study Quality

The recommended Strengthening the Reporting of Observational Studies in Epidemiology (STROBE) [20] checklist was used to assess the quality in all identified and collected full-text articles included in this study (Table 1). The quality of the included studies was assessed independently by two authors (M.H. and E.A.).

Twenty-two checklist criteria were selected, and the collected studies were classified into three categories: studies presenting 19 out of 22 criteria were selected as having a low risk of bias, 18–15 criteria were considered as having a moderate risk of bias, and studies which had less than 15 criteria were selected as having a high risk of bias.

The value of weighted kappa statistic between author agreements was 87%. After confirming the quality of each study, two authors independently extracted the data from each selected study. Disagreements were resolved by discussion and by a third person (M.J.) if the two reviewers could not reach a consensus.

### 2.6. Certainty of the Level of Evidence

The GRADE tool (Grading of Recommendations, Assessment, Development, and Evaluation) [31] was used to assess the quality of a body of evidence. The quality of evidence was rated per outcome into one of four categories (high, moderate, low, and very low) (Table 2).

### 2.7. Risk of Bias (RoB) Assessments

Studies that met the inclusion criteria were assessed using The Joanna Briggs Institute Critical Appraisal tools for JBI Systematic Reviews [32]. The tool focuses on method selection, sufficient demographics, presentation, diagnosis, and proper intervention. Studies scoring 4 were deemed as having a “high quality”, scoring 3 was “moderate quality”, and scores of ≤2 were “poor quality”.

### 2.8. Data Analysis

Data analysis was performed using the comprehensive meta-analysis software, version 4, (CMA-V4). Pooled prevalence and 95% confidence intervals (CI) were estimated using the random-effects model. Heterogeneity among studies was assessed using Cochran’s Q statistics, I-squared statistic, and the tau square (T^2^) test. Sensitivity analysis was conducted by removing one study at a time to evaluate its impact on the overall results. Heterogeneity among the included studies was assessed using the chi-squared test and the I-squared statistic. A funnel plot was used to assess potential publication bias, with a symmetrical distribution indicating no significant bias.

## 3. Results

### 3.1. Search of Literature

**Figure 1:**
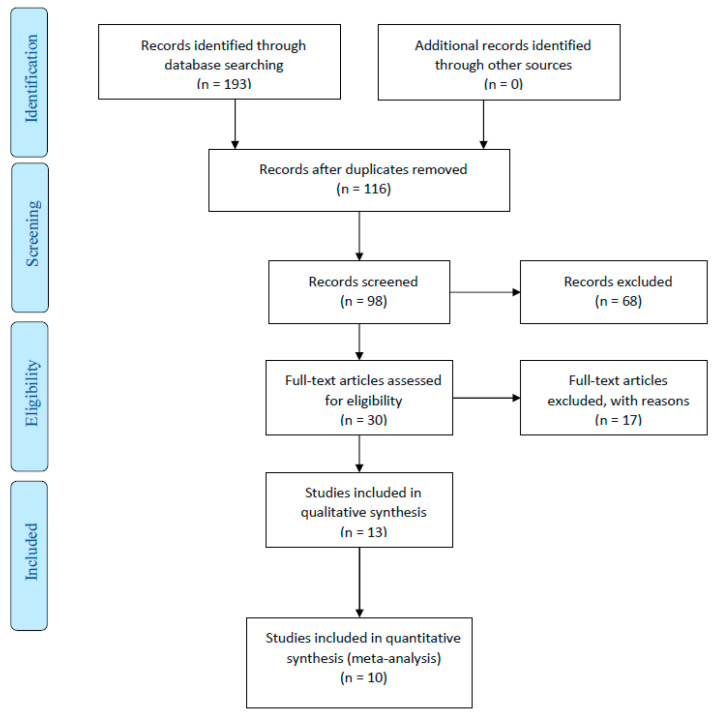
PRISMA flow diagram.

The search strategy yielded a total of 43 articles from all databases. Of the 43 articles, 13 were excluded after an examination of the titles and abstracts, and full-text articles of the remaining 30 studies were reviewed independently for eligibility. Of these 30 studies, 20 were excluded because they did not meet the inclusion criteria. Finally, a total of 10 studies met the inclusion criteria and were processed for critical review (Figure 1). The initial examiners’ average agreement (kappa score) on quality assessment was 0.83. The agreement reached 1.00 after discussion and consensus.

### 3.2. Risk of Bias (RoB) Assessments

In the risk of bias assessments using JBI tools for Systematic Reviews, 8 studies were scored as high quality and 2 scored as moderate quality. Moderate scores were due to a lack of data on some criteria (Table 3).

### 3.3. Study Characteristics

A total of 10 studies that met the inclusion criteria were selected for critical review [21,22,23,24,25,26,27,28,29,30]. The sample size was 902 cases, which were analyzed using descriptive statistics and evaluated based on variables such as frequency and proportions. In total, 902 patients were diagnosed with ACC with an age range of 44.3 [27] to 63 [24] years, and an average age of 56.6 years. Concerning gender, out of 902 patients studied, women were more affected, accounting for up to 56.6% of the patients. The female to male ratio ranges from 1:1 [25] to 2.4:1 [23]. Regarding the primary site of ACC, the palate was the most common location, accounting for 30.5% [29] to 83.3% [24], followed by the buccal mucosa, 4.1% [28] to 33.3% [25], floor of the mouth, 8.3% [28] to 35.2% [23], and lastly, lip and the retromolar area.

For histology, the solid mass pattern was the most prevalent, seen in 95.2% of patients, followed by the cribriform pattern in 4.8% of cases. Regarding treatment modalities, surgery was the most common approach, applied in 76.3% of cases, with a combination of surgery and radiotherapy used in 29.0% of cases (Table 4 and Table 5). A smaller fraction, 3.2%, received a combination of surgery, chemotherapy, and radiotherapy, and 8.3% underwent radiotherapy alone. In terms of disease control and survival among cases with available data, 79.2% demonstrated no evidence of disease. Local recurrence rates varied between 1% [23] and 28.5% [24], and distant metastasis occurred in 18.2% [30] to 33.3% [25] of cases, predominantly to lymph nodes (14.5%) [21]. An analysis of overall survival across various stages and patient numbers indicated a 5-year survival rate of 68.0% among 398 patients.

### 3.4. Meta-Analysis

A meta-analysis was conducted to summarize the findings of various studies investigating different aspects of ACC (Table 6). Ten studies were included in the analysis. The mean age of affected patients was 56.68 years (95% CI: 53.42–59.9 years), and there was a predominance of females. The most prevalent clinical feature of ACC was painless swelling (81.41%, 95% CI: 58.43% to 93.17%), followed by oral ulceration (14.9%). The most common sites of ACC were the hard palate (47.54%), buccal mucosa (24.05%), and tongue (22.18%), while the retromolar area had the lowest prevalence (10.7%). Heterogeneity among studies ranged from moderate to substantial (I2 values: 30.66% to 90.99%). The overall effects were significant for all features except the hard palate, indicating a significant association between clinical features and ACC. There was no significant publication bias based on Egger’s test (*p*-values: 0.0049 to 0.8864) in the included studies.

## 4. Discussion

ACC is a rare malignancy with limited available data on treatment and survival outcomes. The objective of this systematic review was to provide a comprehensive understanding of ACC in minor salivary glands and its clinical features, treatment patterns, and survival outcomes. The findings of this study contribute to the existing literature by synthesizing the available evidence and highlighting key aspects of ACC management. The prevalence of ACC varies across different geographical regions, and in some studies, it was identified as the most common malignant tumor in certain areas. For example, a study from Southern Poland highlighted ACC as a leading malignant salivary gland tumor in the region [33]. Contrary to the findings in some European centers, the WHO designates mucoepidermoid carcinoma as the most common malignant salivary gland tumor [34]. This discrepancy might stem from variations in demographic factors.

The average age of patients in this study aligns with previous reports, indicating that ACC commonly affects individuals in their late forties [35,36,37,38,39,40,41]. The extended time span between symptom onset and diagnosis observed in this study aligns with most case series, although it differs from a few reports [35,42,43]. This discrepancy could be attributed to the slow-growing nature of ACC and its manifestation through nonspecific signs.

The gender distribution of ACC in minor salivary glands shows a female predominance; female to male ratios vary across different studies, such as 2.4:1 in Kusama et al. [23] and 2:1 in Buchner et al. [28]. This is consistent with recent literature, where some studies have reported a higher incidence in women, although the reason for this gender bias remains unclear [44].

The current review encompassed data from different countries like the USA, Japan, Australia, Brazil, Libya, China, and Greece. Such variations may hint at regional differences in etiology, such as genetic factors, environmental exposures, or lifestyle choices. A review by Coca-Pelaz et al. [1] highlighted the importance of understanding regional variations in the presentation of ACC.

In the current review, the most common clinical feature of intraoral ACC is painless swelling. This symptom aligns with the findings in contemporary literature, where painless masses have been reported as a common initial sign [45,46,47,48,49,50,51,52]. The underlying pathophysiology, although not entirely clear, may be attributed to the slow-growing nature of the tumor. Ulceration is also a notable clinical feature in the present study. Ouyang et al. [22] reported painless ulceration in 3.5% of the cases. This may be a sign of the local aggressiveness of the tumor or associated with advanced-stage disease [53]. It is important to note that variations in symptom reporting may be due to the small sample sizes of previous studies, highlighting the need for larger-scale investigations. Some studies have noted the occurrence of pain, despite ACC being predominantly a painless swelling. Pain and other neurological symptoms might be linked to PNI, a characteristic feature of ACC. Hyam et al. [25] reported PNI in 60% of cases. This pattern supports findings in the broader literature, highlighting the complex nature of pain in ACC [54].

The location of ACC in minor salivary glands varies, with a strong predilection for the hard palate. The studies included in the review show palatal involvement with a range from 30.59% in Tian et al. [29] to 79.17% in Bardwil et al. [21]. Other locations include the buccal mucosa, floor of the mouth, and upper lip. The varied distribution may be reflective of the widespread distribution of minor salivary glands [55].

There are three main histopathological patterns for ACC: cribriform, tubular, and solid. All these subtypes can be identified based on the dominant shape and arrangement of the epithelial secreting cells, the myoepithelial cells, and the extracellular matrix. Histologically, a tumor is classified as the solid subtype when this pattern comprises over 30% of the tumor [56]. A study analyzing 87 cases of ACC found the cribriform pattern to be the most prevalent, with the solid form being the rarest [57]. These patterns were used to assess the clinicopathological and prognostic attributes of each subtype. It was observed that the solid subtype had the least differentiated cells, denser extracellular stroma, and was highly locally aggressive, showing the greatest frequency of PNI and the poorest prognosis. Contrarily, Belulescu et al. reported that 46% of their cases showed the solid pattern, challenging the previously observed rarity of this subtype [58]. Such discrepancies might be due to factors like study sample, methodology, and population demographics. Another significant histological feature in ACC is PNI, which has been closely associated with distant metastasis and adverse disease outcomes [59,60]. Additionally, studies focusing on immunohistochemistry labeling have indicated that in ACC, the proliferation and differentiation of myoepithelial cells significantly contribute to the progression of carcinogenesis, more so than the epithelial/secreting cells [61].

PNI is a hallmark feature of ACC, occurring in up to 80% of cases [62]. It is a phenomenon where cancer cells invade the surrounding nerves, leading to a more complex clinical presentation. PNI in ACC has been linked with local recurrence, distant metastasis, and reduced overall survival [63]. The presence of PNI may necessitate more aggressive treatment approaches and has been considered as a predictor of disease progression and unfavorable outcomes [64].

Treatment for ACC is multifaceted, encompassing surgery, radiation therapy, and chemotherapy, tailored to the specific histological pattern and presence or absence of PNI. However, surgical intervention remains the principal method for addressing ACCs that originate from both major and minor salivary glands in the head and neck region. In a systematic review, Ran and coworkers [65] examined current treatment strategies for ACC and reported that surgery alone was the primary treatment for over 40% of cases. Combined surgery and postoperative radiotherapy were used in 35% of cases, while standalone radiotherapy was administered in 19%. This study specifically focused on evaluating the efficacy of the two predominant treatment modalities: surgery only and surgery supplemented with postoperative radiotherapy. The findings indicated superior 5-year and 10-year survival rates for the combination of surgery and postoperative radiotherapy, with respective survival rates of 97.3% and 44.4%, compared to 86.4% and 55.6% for surgery alone. Furthermore, another systematic review explored the role of elective neck dissection in conjunction with surgery in managing ACC. This review revealed that patients who underwent surgery combined with elective neck dissection experienced longer periods without metastasis. However, it was advised that elective neck dissection should be restricted to the first three levels of the lymph nodes to optimize outcomes [66]. Additionally, Su and Yang [67] in a systematic review addressed the outcomes of surgical intervention for ACC metastases in the lungs, underscoring that the surgical excision of metastatic lesions can impede disease progression and enhance overall survival. The success of this approach, however, is contingent on various factors, including the patient’s lung health, the size of the metastases, and the patient’s overall physical condition. Radiotherapy as a standalone treatment is rarely employed for ACC due to its limited effectiveness. It is typically reserved for advanced and inoperable cases of ACC [68]. In contrast, the application of postoperative radiotherapy in conjunction with surgery is acknowledged as a beneficial approach and has gained widespread acceptance in ACC management [69]. Notably, studies have shown that patients who did not receive postoperative radiotherapy were 13 times more susceptible to local recurrence compared to those who underwent the treatment [70]. Chemotherapy as a standalone intervention has shown limited efficacy in the treatment of ACC. This has been evidenced through a range of clinical trials that have assessed various chemical compounds for their potential as systemic therapies. This situation underscores the critical necessity for more comprehensive research in the clinical, pathological, and genetic domains. Such research endeavors are pivotal for a deeper understanding of the processes involved in the carcinogenesis and pathogenesis of ACC. They are also crucial in the quest to discover and develop innovative therapeutic approaches that directly address the root causes and mechanisms of this form of cancer. 

The effectiveness of systemic molecular treatments in advanced ACC has been documented. Initial trials using multi-kinase inhibitors like sunitinib did not demonstrate significant response rates, although most patients experienced disease stability and moderate toxicity [71]. When mTOR was targeted, neither complete nor partial responses were seen, but the median time before disease progression was 11.2 months, and tumor reduction was noted in 44% of the patients [72]. Furthermore, the use of the multi-kinase inhibitor lenvatinib led to partial responses in 5 out of 33 patients, with 75% (24 patients) showing disease stability, indicating that molecular target therapy holds potential [73].

A significant portion of the literature focusing on ACC in the head and neck does not discuss the status of the margins. Garden et al. [74] reported that of 198 patients, 42% had microscopically positive margins, while another 28% presented with margins that were either close or ambiguous. In Erovic et al.’s research [75], positive surgical margins were detected in 60% of ACC cases. Lee et al. [76] documented a 50.82% occurrence of tight or positive surgical margins.

The findings of this study indicated that a 5-year survival rate of patients undergoing surgery followed by radiotherapy was 68.0%. Postoperative radiation therapy is generally recommended for all patients with ACC of the minor salivary glands, regardless of the extent of resection [59,60].

The high frequency of relapses and resistance to therapy in ACC has been connected to both its tendency for topical and regional invasion, and its property of PNI [77]. The common malignant feature of ACC is distant metastasis. 

ACC is characterized by both local and systemic dissemination to various organs. In this study, local and distant metastases were reported among 18.2% [30] to 22.9% [21] of the cases, especially in the lymph nodes and lungs. In a retrospective analysis of patients with distant metastases, the lungs were identified as the exclusive site of metastasis in the majority of the cases [78]. Further, cervical lymph node involvement is seen in approximately 14.5% of ACC patients, as evidenced by a large-scale retrospective study in China, which documented cervical lymph node metastasis in 10% of 798 ACC cases [79]. Multiple studies have also reported the liver as a primary site for systemic disease spread. While metastasis to the breast and larynx is less frequent, extremely rare instances of metastasis have been noted in areas such as the pituitary gland, sternum, dorsal spine [80], choroid, bones of the toe, and the pericardium [80,81,82,83,84,85,86]. Recurrences in ACC are frequent, and in post-initial treatment, the chances of reoccurrence can be up to 50% in particular cases [77]. This significant likelihood of reoccurrence, coupled with the absence of thorough and effective treatment, leads to an unfavorable prognosis. The resistance of recurrent and metastasized ACC to treatment over lengthy durations further complicates disease control. Research has shown a more favorable prognosis in younger patients and females, with survival rates of 90.34%, 79.88%, and 69.22% at 5, 10, and 15 years, respectively [87]. However, a 2020 study with a small sample size of 49 participants reported overall survival rates varying between 68% to 80% for 5 years and 52% to 65% for 10 years, with long-term survival rates ranging from 23% to 40% [88]. The combination of PNI with locoregional invasion has been identified as significant risk factors that contribute to recurrence and resistance to therapy, with risks rising sharply when these factors are present together [77]. The management and prognosis of recurrent and metastasized ACC that results from PNI and conventional spread are often intricate and difficult. Such cases might necessitate multiple surgeries and subsequent radiotherapy after resection. Furthermore, both recurrence and metastasis are frequently linked with adverse long-term prognosis and a lack of disease-free survival [89]. Moreover, even in patients where ACC was diagnosed and resected in the early stage, there is a high risk of distant metastases, particularly in patients with age >45 years, lymph node involvement, and high-grade solid subtype histological features [90]; thus, a follow-up period of approximately 15 years with annual chest CT scans with contrast post-treatment is recommended.

The continued study of ACC’s molecular biology may lead to new targeted therapies. Furthermore, developing refined imaging techniques for better assessment of PNI and improving early detection will be critical in advancing ACC management [91].

Several limitations should be considered when interpreting the findings of this study. The included studies varied in quality, with inconsistent reporting of observational variables. The rarity of ACC and limited data availability result in lower levels of evidence. The wide time span covered by the studies may have influenced outcome results due to advancements in technology, terminology, and treatment. Furthermore, within this systematic review, the work conducted by Tian et al. [29] stands out as the largest, comprising 681 cases out of the total cases examined. As a result, it is crucial to approach the interpretation of this study’s findings with caution. The potential for variations in diagnosis and reporting has the potential to influence the accuracy and dependability of the study’s conclusions. Consequently, while the study offers invaluable insights, researchers and clinicians must recognize the inherent limitations and exercise caution when applying its findings. Nevertheless, the findings of this study provide valuable insights for physicians in making treatment decisions and emphasize the need for ongoing research and collaborative clinical efforts to improve the management and outcomes of this challenging disease.

## 5. Conclusions

ACC of the minor salivary glands is a multifaceted condition typically manifesting as asymptomatic enlargement and ulceration. This disease is marked by distinct histopathological patterns and PNI. Recognizing these distinctive aspects is key in shaping the treatment plan, which can range from surgical procedures to radiation therapy, chemotherapy, and evolving targeted treatments. Continuous research and collaborative clinical efforts remain critical for ongoing progress in the treatment and management of this challenging condition.

## Figures and Tables

**Table 1:** Strobe Quality Assessment.

Author/ Year	Title/ Abstract	Background	Objectives	Study Design	Setting	Participants	Variables	Data/ Sources	Bias	Study Size	Quantitative Variables	Statistical Method	Descriptive Data	Outcome Data	Main Results	Other Analysis	Key Results	Limitations	Interpretation	Generalizability	Funding
Bardwil et al. 1966 [21]	Yes	Yes	Yes	Yes	Yes	Yes	Yes	Yes	No	Yes	Yes	Yes	Yes	Yes	Yes	No	Yes	Yes	Yes	Yes	Yes
Charles et al. 1988 [22]	Yes	Yes	Yes	Yes	Yes	Yes	Yes	Yes	No	Yes	Yes	Yes	Yes	Yes	Yes	No	Yes	Yes	Yes	Yes	Yes
Kusama et al. 1997 [23]	Yes	Yes	Yes	Yes	Yes	Yes	Yes	Yes	No	Yes	Yes	Yes	Yes	Yes	Yes	No	Yes	Yes	Yes	Yes	Yes
Jansisyanont et al. 2002 [24]	Yes	Yes	Yes	Yes	Yes	Yes	Yes	Yes	No	Yes	Yes	Yes	Yes	Yes	Yes	No	Yes	Yes	Yes	Yes	Yes
Hyam et al. 2004 [25]	Yes	Yes	Yes	Yes	Yes	Yes	Yes	Yes	No	Yes	Yes	Yes	Yes	Yes	Yes	No	Yes	Yes	Yes	Yes	Yes
Ito et al. 2005 [26]	Yes	Yes	Yes	Yes	Yes	Yes	Yes	Yes	No	Yes	Yes	Yes	Yes	Yes	Yes	No	Yes	Yes	Yes	Yes	Yes
Jaber 2006 [27]	Yes	Yes	Yes	Yes	Yes	Yes	Yes	Yes	No	Yes	Yes	Yes	Yes	Yes	Yes	No	Yes	Yes	Yes	Yes	Yes
Buchner et al. 2007 [28]	Yes	Yes	Yes	Yes	Yes	Yes	Yes	Yes	No	Yes	Yes	Yes	Yes	Yes	Yes	No	Yes	Yes	Yes	Yes	Yes
Tian et al. 2010 [29]	Yes	Yes	Yes	Yes	Yes	Yes	Yes	Yes	No	Yes	Yes	Yes	Yes	Yes	Yes	No	Yes	Yes	Yes	Yes	Yes
Triantafillidou et al. 2006 [30]	Yes	Yes	Yes	Yes	Yes	Yes	Yes	Yes	No	Yes	Yes	Yes	Yes	Yes	Yes	No	Yes	Yes	Yes	Yes	Yes

**Table 2:** Certainty of the level of evidence.

Author/Year	Limitation	Inconsistency	Indirectness	Imprecision	Publication Bias	Overall Level of Evidence
Bardwil et al. 1966 [21]	No serious limitation	No serious inconsistency	No serious indirectness	No serious imprecision	No serious publication bias	High
Charles et al. 1988 [22]	No serious limitation	No serious inconsistency	No serious indirectness	No serious imprecision	No serious publication bias	High
Kusama et al. 1997 [23]	No serious limitation	No serious inconsistency	No serious indirectness	No serious imprecision	No serious publication Bias	High
Jansisyanont et al. 2002 [24]	No serious limitation	No serious inconsistency	No serious indirectness	No serious imprecision	No serious publication bias	High
Hyam et al. 2004 [25]	No serious limitation	No serious inconsistency	No serious indirectness	No serious imprecision	No serious publication bias	High
Ito et al. 2005 [26]	No serious limitation	No serious inconsistency	No serious indirectness	No serious imprecision	No serious publication bias	High
Jaber 2006 [27]	No serious limitation	No serious inconsistency	No serious indirectness	No serious imprecision	No serious publication bias	High
Buchner et al. 2007 [28]	No serious limitation	No serious inconsistency	No serious indirectness	No serious imprecision	No serious publication bias	High
Tian et al. 2010 [29]	No serious limitation	No serious inconsistency	No serious indirectness	No serious imprecision	No serious publication bias	High
Triantafillidou et al. 2006 [30]	No serious limitation	No serious inconsistency	No serious indirectness	No serious imprecision	No serious publication bias	High

**Table 3:** Risk of Bias Assessment.

Author/Year	1. Were There Clear Criteria for Inclusion in the Case Series?	2. Was the Condition Measured in a Standard, Reliable Way for All Participants Included in the Case Series?	3. Were Valid Methods Used for Identification of the Condition for All Participants Included in the Case Series?	4. Did the Case Series Have Consecutive Inclusion of Participants?	5. Did the Case Series Have Complete Inclusion of Participants?	6. Was There Clear Reporting of the Demographics of the Participants in the Study?	7. Was There Clear Reporting of Clinical Information of the Participants?	8.Were the Outcomes or Follow-Up Results of Cases Clearly Reported?	9. Was There Clear Reporting of the Presenting Sites’/Clinics’ Demographic Information?	10. Was Statistical Analysis Appropriate?	Quality Score
Bardwil et al. 1966 [21]	Yes	NA	Yes	Yes	Yes	Yes	Yes	Yes	Yes	Yes	High quality
Charles et al. 1988 [22]	Yes	NA	Yes	Yes	Yes	Yes	Yes	Yes	Yes	Yes	High quality
Kusama et al. 1997 [23]	Yes	NA	Yes	Yes	Yes	Yes	Yes	Yes	Yes	Yes	High quality
Jansisyanont et al. 2002 [24]	Yes	NA	Yes	Yes	Yes	Yes	Yes	Yes	Yes	Yes	High quality
Hyam et al. 2004 [25]	Yes	NA	Yes	Yes	Yes	Yes	Yes	Yes	Yes	Yes	High quality
Ito et al. 2005 [26]	Yes	NA	Yes	Yes	Yes	Yes	Yes	Yes	Yes	Yes	High quality
Jaber 2006 [27]	Yes	NA	Yes	Yes	Yes	Yes	Yes	Yes	Yes	Yes	High quality
Buchner et al. 2007 [28]	Yes	NA	Yes	Yes	Yes	Yes	Yes	Yes	Yes	Yes	High quality
Tian et al. 2010 [29]	Yes	NA	Yes	Yes	Yes	Yes	Yes	Yes	Yes	Yes	High quality
Triantafillidou et al. 2006 [30]	Yes	NA	Yes	Yes	Yes	Yes	Yes	Yes	Yes	Yes	High quality

**Table 4:** Characteristics of the included studies.

Author/Year	Study Type	Country	No. of Cases	Mean Age	M/F Ratio	Clinical Feature Mass/Ulcer/Pain	Main Location
Bardwil et al. 1966 [21]	Retrospective	USA	48	48.5 years	-	No features (35.42%) Painless swelling (41.67%) No ulceration (22.92%)	Hard palate (79.17%) Lip (20.83%)
Charles et al. 1988 [22]	Retrospective	USA	40	57.7 ± 17.59 years	F/M ratio = 1.5/1	Painless ulceration (87.5%) Swelling (12.5%)	Hard palate (47.5%) Upper lip (5%) Buccal mucosa (17.5%) Floor of the mouth and retromolar (22.5%)
Kusama et al. 1997 [23]	Retrospective	Japan	17	55.1 ± 15 years	F/M ratio = 2.4/1	Painless swelling (100%)	Hard palate (41.18%) Retromolar (5.88%) Buccal mucosa (17.65%) Floor of the mouth (35.29%)
Jansisyanont et al. 2002 [24]	Retrospective	USA	6	63 years	F/M ratio = 1.9/1	Painless swelling (100%)	Hard palate (83.3%) Upper lip (16.7%)
Hyam et al. 2004 [25]	Retrospective	Australia	12	62 years	Male = 6 Female = 6	Pain and swelling (100%)	Hard palate (58.33%) Buccal mucosa (33.33%) Oropharynx (8.33%)
Ito et al. 2005 [26]	Retrospective	Brazil	39	57 years	Male = 19 Female = 20	Not mentioned	Palate (67.5%) Other (7.7%)
Jaber 2006 [27]	Retrospective	Libya	13	44.3 years	F/M ratio = 1/0.7	Swelling (38.46%) Ulcer (30.77%) Pain (15.38%) Ill-fitting prosthesis (15.38%)	Palate (38.46%) Tongue (23.08%) Other (38.46%)
Buchner et al. 2007 [28]	Retrospective	USA	24	57 years	F/M ratio = 2:1	Painless swelling (100%)	Hard palate (75%) Upper lip (8.3%) Floor of the mouth (8.3%) Buccal mucosa (4.17%) Retromolar region (4.17%)
Tian et al. 2010 [29]	Retrospective	China	681	51.45 years	F/M ratio = 1.25/1	Swelling (80–90%)	Hard palate (30.59%) Parotid (11.6%) Submandibular (10.87%) Others (46.84%)
Triantafillidou et al. 2006 [30]	Retrospective	Greece	22	22–87	1.2:1	Swelling (100%)	Palate (18%) Buccal mucosa (3%) Floor of the mouth (1%)

**Table 5:** Histology and treatment outcomes of ACC.

Author/Year	Histological Pattern	Perineural Invasion	Treatment Received	Treatment Margins	Follow-Up Duration	Metastasis/ Recurrence	Location of Metastasis
Bardwil et al. 1966 [21]	Not mentioned	Yes (45.83%)	1. Surgical excision and en bloc resection (77.08%) 2. Excision followed by radiation (14.58%) 3. Radiation only (8.33%)	90% negative 10% positive	3 years	Distant metastasis (22.92%) Recurrence (20.83%)	Lymph nodes (14.58%) Intracranial (8.33%)
Charles et al. 1988 [22]	Cribriform appearance (80%) Solid (5%) Tubular (15%)	No	Not mentioned	Not mentioned	Not mentioned	No metastasis Recurrence not reported	Not applicable
Kusama et al. 1997 [23]	Solid (100%)	No	Not mentioned	Not mentioned	16 years	Local metastasis Recurrence (1%)	Cervical lymph nodes
Jansisyanont et al. 2002 [24]	Not mentioned	No	Wide local excision (70.8%) Surgery + radiation + chemotherapy (18%) Chemotherapy and/or radiation (3.2%) Other (8%)	100% negative	7 years, 6 months	Distant metastasis Recurrence (28.57%)	Cervical lymph nodes, lung, bone, and brain (16.67%)
Hyam et al. 2004 [25]	Solid mass	Yes (60%)	Surgery as the primary modality (33.33%) Patients with a positive surgical margin were recommended adjuvant radiotherapy (66.67%)	47% positive surgical margin 10% close margin (<2 mm) negative	5 years	Metastasis (33.3%) Recurrence (8.3%)	Bone
Ito et al. 2005 [26]	Not mentioned	No	Surgical excision (100%)	100% negative	29 years	No metastasis Recurrence not reported	Not applicable
Jaber 2006 [27]	Not mentioned	No	Surgery or radiotherapy alone or a combination of both	Not mentioned	7 years	No metastasis Recurrence (15.3%)	Not applicable
Buchner et al. 2007 [28]	Solid mass	No	Not mentioned	Not mentioned	20 years	Not mentioned Recurrence not reported	Not applicable
Tian et al. 2010 [29]	Solid mass	No	Not mentioned	Not mentioned	23 years	No metastasis Recurrence not reported	Not applicable
Triantafillidou et al. 2006 [30]	Solid mass	Yes	Surgery as the primary modality (100%) Surgery + radiation, 17 patients	Not mentioned	21 years	Metastasis 4 cases (18.2%) Recurrence (9.1%)	Lung (9.1%) Lymph nodes (9.1%)

**Table 6:** Meta-analysis of the selected studies.

Demographical Characteristics									
Age (mean in years)									
Female	10	56.68	53.42–59.9	9	5.13	0	0	<0.001	0.0677
**Clinical features**									
Painless swelling	10	81.41	58.43–93.17	9	99.95	90.99	2.333	<0.001	0.8864
Ulceration	10	14.9	0.6–3.22	9	63.67	85.86	1.63	0.0262	0.7274
Hard palate	10	47.54	32.98–62.53	9	64.19	85.98	0.73	0.0921	0.0921
Lip	7	16.97	11.73–23.91	6	10.96	45.24	0.12	0.0897	0.5506
Buccal mucosa	9	24.05	16.21–34.13	8	25.39	68.49	0.30	0.0013	0.0049
Floor of mouth	8	17.82	10.81–27.94	7	23.35	70.02	0.39	0.0015	0.4604
Retromolar	3	10.7	0.04–24.99	2	2.88	30.66	0.29	0.2364	0.0997
Tongue	5	22.18	7.2–51.15	4	18.57	78.46	1.46	0.0009	0.6772

Q Cochran’s Q statistic for heterogeneity. I^2^ index for the degree of heterogeneity. T^2^ tau-squared measure of heterogeneity.

## Data Availability

Not applicable.
